# Evaluation of a Mobile Device Survey System for Behavioral Risk Factors (SHAPE): App Development and Usability Study

**DOI:** 10.2196/10246

**Published:** 2019-01-11

**Authors:** Ingrid Oakley-Girvan, Juan M Lavista, Yasamin Miller, Sharon Davis, Carlos Acle, Jeffrey Hancock, Lorene M Nelson

**Affiliations:** 1 Public Health Institute Oakland, CA United States; 2 Canary Center at Stanford for Cancer Early Detection Stanford University School of Medicine Stanford, CA United States; 3 Stanford Cancer Institute Stanford University Stanford, CA United States; 4 Microsoft Kirkland, WA United States; 5 Yasamin Miller Group Ithaca, NY United States; 6 Department of Research Cancer Prevention Institute of California Fremont, CA United States; 7 Onetree Kirkland, WA United States; 8 Department of Communication Stanford University Stanford, CA United States; 9 Stanford Center for Population Sciences Department of Health Research and Policy Stanford University School of Medicine Stanford, CA United States

**Keywords:** mobile phone, demographic characteristics, health surveys, risk behaviors, mobile apps

## Abstract

**Background:**

Risk factors, including limited exercise, poor sleep, smoking, and alcohol and drug use, if mitigated early, can improve long-term health. Risk prevalence has traditionally been measured using methods that now have diminished participation rates. With >75% of American citizens owning smartphones, new data collection methods using mobile apps can be evaluated.

**Objective:**

The objective of our study was to describe the development, implementation, and evaluation of a mobile device–based survey system for behavioral risk assessment. Specifically, we evaluated its feasibility, usability, acceptability, and validity.

**Methods:**

We enrolled 536 students from 3 Vermont State Colleges. Iterative mobile app development incorporated focus groups, extensive testing, and the following 4 app versions: iOS standard, iOS gamified, Android standard, and Android gamified. We aimed to capture survey data, paradata, and ambient data such as geolocation. Using 3 separate surveys, we asked a total of 27 questions that included demographic characteristics, behavioral health, and questions regarding the app’s usability and survey process.

**Results:**

Planned enrollment was exceeded in just a few days. There were 1392 “hits” to the landing page where the app could be downloaded. Excluding known project testers and others not part of the study population, 670 participants downloadeded the SHAPE app. Of those, 94.9% of participants (636/670) agreed to participate by providing in-app consent. Of the 636 who provided consent, 84.3% (536/636) were deemed eligible for the study. The majority of eligible respondents completed the initial survey (459/536, 85.6%), whereas 29.9% (160/536) completed the second survey and 28.5% (153/536) completed the third survey. The SHAPE survey obtained 414 participants on the behavioral risk items in survey 1, which is nearly double the 209 participants who completed the traditional Vermont College Health Survey in 2014. SHAPE survey responses were consistent with the traditionally collected Vermont College Health Survey data.

**Conclusions:**

This study provides data highlighting the potential for mobile apps to improve population-based health, including an assessment of recruitment methods, burden and response rapidity, and future adaptations. Although gamification and monetary rewards were relatively unimportant to this study population, item response theory may be technologically feasible to reduce individual survey burden. Additional data collected by smartphones, such as geolocation, could be important in additional analysis, such as neighborhood characteristics and their impact on behavioral risk factors. Mobile tools that offer rapid adaptation for specific populations may improve research data collection for primary prevention and could be used to improve engagement and health outcomes.

## Introduction

Monitoring risk behavior prevalence is critical for public health planning and interventions. Early mitigation of specific risk factors for poor health, such as limited exercise, poor sleep, mood disorders, smoking, and drug and alcohol use, can prevent serious long-term health consequences in the general population. Across the United States, 25% of adults smoke [1]. With only 21% of US adults meeting the recommended levels of physical activity [1], it is not unexpected that over a third (35.8%) are overweight [2]. Alcohol abuse and dependence are highly prevalent [3-5], as are serious mood disorders [6] and illegal drug abuse [7]. Although there are smartphone apps that measure many risk factors [8-12], there is currently no ability to rapidly collate the results of these measurements to determine the population-specific risk prevalence that can be used for public health planning and interventions.

Risk prevalence is currently measured through telephonic surveys using methods such as random digit dialing (RDD) that now faces ever-diminishing participation rates among eligible subjects and bias because of migration from landlines to mobile phones [13]. The Behavioral Risk Factor Surveillance System [14] is the largest RDD survey in the United States with 500,000 annual respondents, but it has suffered a 20% drop in response rates during the past decade [15]. Nonresponse can vary significantly across different demographic and geographic groups. Although procedures to adjust for this nonresponse can be utilized, they may result in diminished statistical precision [16]. As in many other fields (including preelection polling), telephone surveys are increasingly unreliable, and the search for a modern-day alternative is justified to ensure the continuity of valid measurements. Collecting survey data using smartphone apps may offer many advantages, including the potential to collect geopositioning, scanning, photo, and video data. Over 75% of American citizens now own a smartphone [17], and underrepresented groups have often been quick to adopt this technology and forgo landline telephones [18].

Web surveys have been used to collect information on sensitive behaviors. However, the mode of survey administration was found to affect responses for about one-third of variables in one study (the British National Survey of Sexual Attitudes and Lifestyles), which compared a Web survey interview design with computer assisted personal interview and self-interviews [19]. Other criticisms of Web surveys include the lack of research on the effects of format or design on the levels of unit and item response or data quality [20] and the lack of representativeness compared with the general population [21].

Although surveys have been conducted to determine how mobile phone owners use health apps [22,23], few papers provide detail on how to develop a survey app specifically designed to administer a variety of surveys [24]. A framework for developing the survey apps was proposed by Buskirk and Andres [25] who presented an outline of app-based smartphone survey approaches. Davis and Oakley-Girvan [26] provided strategies to improve testing and validation of mobile apps, including iterative testing, enhanced user engagement, reduced burden, and appropriate infrastructure to reduce downtime and meet Health Insurance Portability and Accountability Act (HIPAA) privacy and confidentiality of personal health information requirements.

This study focuses on developing, implementing, and pilot-testing a mobile survey system to collect behavioral risk data from college students and addresses the following questions:

- Feasibility: Can the target population be recruited to download and use the app?
- Usability: Can an app be developed that is easy for people to understand and quick to use?
- Acceptability: Will respondents allow access to phone-captured ambient data?
- Validity: How well do the app data correspond to traditionally collected data from the target population?

The hypothesis was that utilizing an iterative development and testing approach would yield an effective app with low burden and high acceptability to collect behavioral health and demographic characteristics consistent with previous benchmarks. This paper presents the results of this pilot study that addresses the 4 questions provided above.

The objective of this paper was to describe the development and evaluation of a mobile app to administer behavioral health-related surveys on iOS (Apple) and Android platforms with at least 500 pilot users from 3 small northeastern colleges and at least 20 behavioral health-related survey questions. We chose a college population because the characteristics of the entire population were already known through other survey mechanism. Similar survey questions had already been collected on this population through traditional RDD telephone and Web surveys which provided a comparison for our mobile phone app survey results. The evaluation was developed to include a process to encourage individual enrollment by downloading the mobile app, providing a mechanism to invite users to respond to consecutive short surveys within the mobile app, and capitalizing on the ability to pull location from users’ mobile phone.

## Methods

### Study Protocol Summary

The study population included students enrolled in 3 Vermont State Colleges (Castleton University, CU; Lyndon State College, LSC; and Johnson State College, JSC). In the summer of 2016, we conducted a focus group with a convenience sample of CU students (n=9) to elicit suggestions concerning branding, color scheme, and a name for the app (ultimately named “SHAPE”). Based on this input, the study team created 4 versions of the SHAPE app (iOS standard, iOS gamified, Android standard, and Android gamified) to collect behavioral health data. Survey questions focused on the demographic characteristics and behavioral health items consistent with available benchmark data. A multipronged approach was used to recruit student participants during a 22-day period in October 2016. Additional details on recruitment methods are included in the “Participant Recruitment” section. Similar to traditional

telephone surveys, where the informed consent is administered after potential study subjects answer the phone, SHAPE participants were administered the informed consent process and institutional review board approved consent materials embedded within the mobile app download and eligibility determination process. Eligible participants (aged ≥18 years with an email domain at a participating institution) who consented were administered the first survey. Two additional surveys were administered by utilizing a push out mechanism within the app over a period of several weeks. A total of 27 questions were asked across 3 surveys, the last of which included questions regarding the SHAPE app functioning and the survey process.

A focus group with selected mobile app participants was conducted after app data collection to understand how students liked the app and why they did or did not respond to the surveys. Students (n=7) were recruited in person by the on-campus recruiter, and 8 open-ended questions were discussed.

### App Development

To design and build this multiple component platform, our app development team included user interface (UI) designers, user experience (UX) designers, iOS and Android Developers, Web and backend developers, and Quality Assurance analysts. Utilization of the nimble mobile app platform by Medable and app development team was essential because the rest of the multidisciplinary team did not need to be familiar with the platform and programming language. We were able to successfully create the Council of State and Territorial Epidemiologists SHAPE app on both the iOS and Android operating systems. To meet the objectives of the project, it was necessary to develop an app for each platform (iOS and Android), each having 2 versions—the standard version (only the survey items) and a gamified version (the survey items plus a point system). All 4 versions of the SHAPE app were developed using the native programing languages of each platform, Swift (for iOS) and Android Studio (for Android), and were compatible with iOS 8+ and Android 4.x+.

Native components for both platforms were used to improve the performance and user experience for conducting research. Research Kit was used for iOS, and Research Stack was used for Android; these kits provided a user experience in compliance with the iOS and Android standards as required when submitting research that is included as part of an app. The gamified version was a slightly modified variation of the standard version, which allowed the reuse of substantial portions of app code for both gamified and nongamified versions, enabling the adherence to the time and budget limitations of this project. Owing to the use of native technologies, there were minor visual design differences between the 2 operating systems. The differences were based on typical design displays for each operating system; for example, Android answer options are displayed with a radio button that is filled in when tapped, whereas iOS users see a checkmark appear to the right of an answer option when tapped. Appendices provide screenshots of both operating systems for comparisons. Of note, any potential differences in subjects’ responses because of these visual design features were not explored.

The backend, the technology component where the data are managed and stored, was another key component of the solution. The backend functionality was provided by Medable and, in particular, by its Axon product, aimed at facilitating the execution of studies using mobile apps. From the backend, the researchers were able to manage users and create studies (surveys) as well as questions and response options. The backend can seamlessly be integrated with the mobile app through the iOS and Android Software Development Kits provided by the Medable platform. As described above, an additional feature implemented in the Medable platform is HIPAA compliance.

Another important aspect of the SHAPE app was the availability of push notifications as a way to notify users that a survey was available and to remind them to participate. These push notification scripts were developed in Medable and executed through the Medable platform to the apps. The Medable backend platform captured responses to the survey items as well as all data related to the game (points) and all paradata (date and time of starting a survey, date and time of ending a survey, geolocation, etc).

### Design of the App

The design of the app was driven by a process that was developed in stages with each stage resulting in the identification of the best intermediate product based on the needs of the overall project and included the development of wireframes, definition of uses cases, UI design, and user experience testing.

#### Development of Wireframes

The start of the development process involved the creation of wireframes and a clear definition of the end product. To gather ideas and get the team discussion moving, it was necessary to first lay out the basic structure and flow of the app, initially with low fidelity paper sketches, followed by digital interactive sketches available on the internet for user testing with the use of Invision (www.invisionapp.com). Through this tool (Invision), the UI/UX team was able to share the progress of the design process and receive feedback and comments directly in the tool from the team members located in different US cities.

#### Definition of Use Cases

In parallel with the wireframes and product specification process, use cases were defined; this development technique was used to explore the potential needs of the end users of the app. As identified through the use cases process, the app brand was an important and challenging requirement of the process. The branding included name creation, logo, and a complete brand guideline that was used across the different components of the platform—apps, dashboard, marketing materials, etc.

#### User Interface Design

Once the flow of both apps (standard and gamified) was defined and wireframes were approved by all team members, we moved to the final UI design stage. For this stage, an interactive prototype was built, and users tested it with the help of Lookback (www.lookback.io), a research tool that captures how users experience the app. The SHAPE brand was tested through interviews with Castleton students. Lookback records user interactions with the product—their screen touches, clicks, and their face and voice. With this information, the UI/UX team was able to observe how users engage with the app, including facial and voice reactions to the app while they are using the app; this gave powerful insight into ways to improve the overall user experience.

#### User Experience Testing

The final stage of the development involved a series of continual tests in which every internal release delivered by the development team was reviewed by the UI/UX team. Improvements were made to UI, obtaining continual feedback to allow the detection of any usability issues that we were unable to test on the prototype. In the final phase of our UI/UX process, the UI/UX designers reviewed the quality of the developed apps, ensuring that the final user experience matched the intended design.

### Survey Questions

Survey questions for which benchmark data were already available from the traditionally administered Vermont College Health Survey (VCHS) [27] included questions related to general health behavior (physical activity and sleep), mental health (depression and stress), and substance use (alcohol, marijuana, tobacco, cocaine, and methamphetamine use). In addition, demographic items (age, gender, race or ethnicity, year in school, and residence) were selected from VCHS. Supplemental questions were developed regarding how respondents learned about the SHAPE app, motivation for participation, evaluation, and “adoptability” of the app [28]. The questions were divided into 3 surveys. Demographic, mental health, and health behavior items (15 items total) were asked in the initial survey. The second survey included questions on substance use (7 items). The final survey asked questions related to evaluating and providing feedback about the app (5 items).

### Marketing or Branding

Student employees of the Castleton Polling Institute created some preliminary names and logos for the app to present to the preapp focus group; this focus group provided information to guide the logo, color scheme, and name selection of the app. Polling Institute student employees helped create general marketing messages and recruitment materials. Prior to the app launch, additional iterative usability research was conducted within the research team and 6 CU students to gather feedback on the app prototype as well as the draft marketing strategy, messages, and recruitment materials.

### Participant Recruitment

A website landing page was created to facilitate easy app download and ensure that participants were randomly assigned to receive either the gamified or standard versions of the app. The landing page included links to the SHAPE app in both Apple’s and Android’s app stores, a frequently asked questions section about the project, a link to the consent form, and contact information for questions or assistance. In addition, Google Analytics and unique links were created to track traffic to the page for each of the recruitment methods. At the launch of the project, approximately 1000 full-color flyers were placed at the 3 campuses in varying locations. Student recruiters were hired at all 3 campuses to encourage participation. Student recruiters were provided leaflets with the landing page link and bookmarks as recruitment aids. CU and JSC published advertisements for the app in their school newspaper, 3 emails were sent to all CU students with links to the landing page, and LSC included recruitment materials in their electronic weekly student newsletter. In the final week of recruitment, targeted Facebook ads were purchased for 5 days at CU. A lunchtime pizza giveaway at JSC and LSC campuses was hosted.

### Implementation: Security and Data Collection

The SHAPE project pilot test was approved by the Institutional Review Boards of Castleton University, Lyndon State College, and Johnson State College, as well as all 3 institutions’ Presidents and the Vermont State College Systems legal counsel. Medable, a medical software development company, provided a HIPAA-compliant backend platform that allowed secure storage and transmission of data and a business associate agreement that meets US Department of Health and Human Services requirements for the protection of human subjects.

As part of the eligibility and consent process, participants were asked their age and institutional email address. A respondent was eligible if they were 18 years of age or older and provided an email address that used a participating institution’s domain. Eligible respondents were asked if they would allow push notifications (messages sent by the SHAPE app to the device but not required) and then given the option to begin the first survey or resume at a later date and time. The first survey remained open during the enrollment period. Any enrolled participant who did not fully complete Survey 1 received push notifications (if they had allowed the notifications on their device) as reminders to complete the survey. A total of 4 push notifications were included during Survey 1’s field period (October 10, 2016-October 31, 2016).

**Figure 1:**
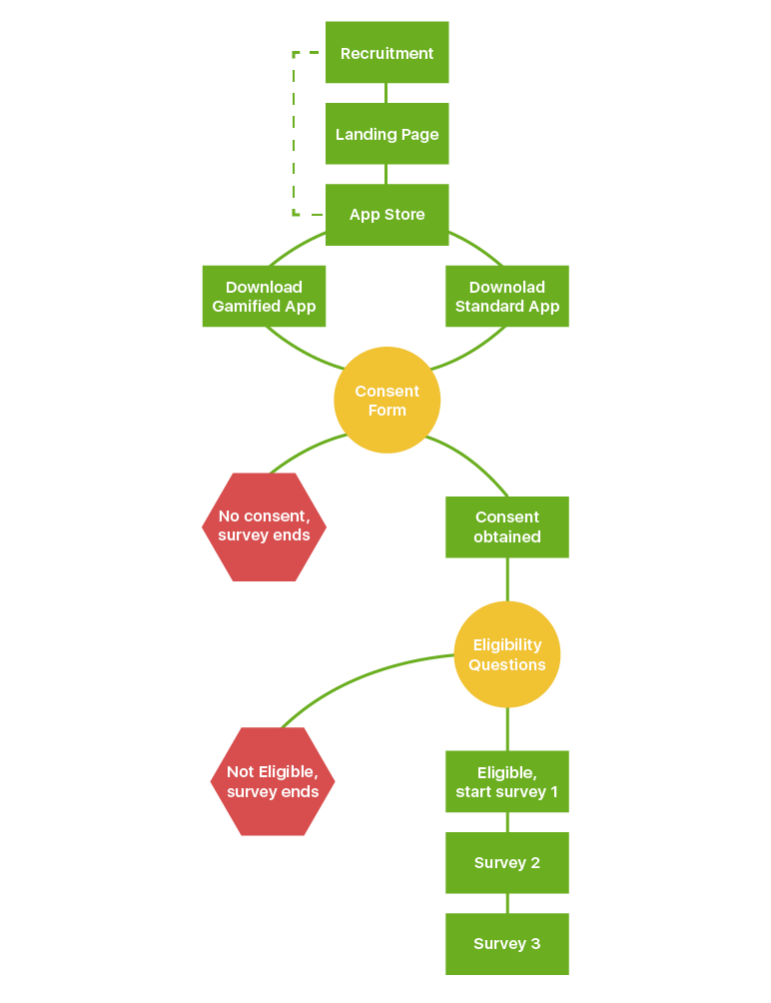
The study protocol schematic.

All eligible respondents, regardless of completion of Survey 1, received a push notification at the start of Surveys 2 and 3 followed by additional reminder push notifications. Because of low initial response to Survey 2 with only push notifications, 3 reminder emails were sent during the second field period (November 17, 2016-November 27, 2016). Survey 3 was launched on November 30, 2016. All enrolled participants, regardless of previous survey completion, were asked to complete Survey 3. The notification protocol included an initial survey push notification, 3 reminder push notifications, and 3 email reminders. Survey 3 closed on December 8, 2016. Figure 1 shows the study protocol from recruitment, consent, eligibility, and survey administration.

### Evaluation of Feasibility, Usability, Acceptability, and Validity

Iterative testing of apps for each platform (iOS and Android) was conducted over 16 weeks. Each app had 2 versions—the standard version (only the survey items) and a gamified version (the survey items plus a simple point system)—with acceptance on the iOS and Android app stores. The SHAPE app was developed using Swift (for iOS) and Android Studio (for Android) and was compatible with iOS 8+ and Android 4.x+. Medable’s Axon product for Research Kit was used for iOS, and Research Stack was used for Android.

From the Medable HIPAA-compliant backend, the researchers were able to manage users, create questions and response options, and push notification scripts. The Medable backend captured real-time responses to the survey items as well as all data related to the game (points) and all paradata (date and time of starting a survey, date and time of ending a survey, geolocation, etc).

Qualitative data (eg, focus groups, UI/UX testing, and interviews) were audiorecorded and reviewed by the project team [29]. Data from the VCHS 2014 survey were compiled in aggregate for the population of interest. Several paradata measures were collected and analyzed [30-33].

## Results

### Feasibility: Participants’ Characteristics

There were 1392 “hits” to the landing page where the app could be downloaded. Excluding known project testers and others not part of the population of study, 670 apps were downloaded. Of those 670, 636 participants (636/670, 94.9%) agreed to participate by providing in-app consent. Of the 636 who consented, 536 (536/636, 84.3%) were deemed eligible for the study. The majority of eligible respondents (459/536, 85.6%) completed Survey 1. Similar to longitudinal studies, the highest rate of attrition occurred between Survey 1 and Survey 2. Retention between surveys 2 and 3 was high because almost equal numbers of respondents to Survey 1 completed Survey 2 (160/459, 34.9%) as we all as Survey 3 (33.3%, 153/459). As reported by Miller et al (Survey Research, In Press 2018), 88.1% (472/536) of eligible respondents were from the primary location (CU), the majority were iOS system users (438/536, 81.7%), and the sample was deemed representative of the target population (Castleton University).

The distribution of years in school was significantly different between completers (those completing all 3 surveys) and noncompleters (those completing only 1 or 2 surveys) with 12.1% more third-year students completing all 3 surveys. The rate of completion between enrolled and eligible participants for all 3 surveys among gamified (76/277) and standard app (77/259) respondents was similar (27.4% vs 29.7%), indicating that the pilot-tested gamified design did not reduce the attrition rate.

### Validity: Comparison to the Benchmark Survey (Vermont College Health Survey)

The data collected from the SHAPE mobile app were compared with the results for CU students obtained from the 2014 VCHS because results from the 2016 survey were not yet available for analysis. Because not all 2014 survey items were the same as the items asked in 2016 (and thus repeated in this project), analyses were restricted to comparisons when the 2016 questions were either identical or had minor differences in wording compared with the 2014 questions. This analysis focused on respondents from the primary site (CU) because of small numbers of participants from JSC and LSC. The SHAPE app items had low item nonresponse rates, similar to response rates from the 2014 VCHS.

A total of 209 CU students responded to the 2014 VCHS. In comparison to enrollment numbers, the age distribution was similar in both VCHS and SHAPE. Women were overrepresented in VCHS data by 22% compared with the 2014 enrollment data, whereas women were only minimally overrepresented (279/536, 2.1%) in the SHAPE app survey respondents. VCHS data included a slightly higher proportion (by 4%) of international students than in total enrollment compared with slightly lower participation in the SHAPE app (−3%). Interestingly, the app improved upon capturing race or ethnicities other than white and had almost double the number of anticipated respondents to survey 1 compared with the 2014 VCHS.

Some examples of health and behavior items that were included in both the 2014 VCHS and the SHAPE app project were as follows:

- On how many of the past 7 days, did you perform moderate-intensity exercise for at least 20 minutes?
- On how many of the past 7 days, did you perform vigorous-intensity exercise for at least 20 minutes?
- On how many of the past 7 days, did you perform exercises to strengthen your muscles (8-10 exercises each for 8-12 repetitions)?
- How often have you used cigarettes?
- How often have you used alcohol?
- During the last [reference period], how many times have you had 5 or more drinks of alcohol at a sitting? (The reference period is 2 weeks in the VCHS and 30 days in SHAPE)
- How often have you used marijuana?
- How often have you used cocaine (eg, crack, rock, blow, and freebase)?
- How often have you used methamphetamine (eg, meth, crystal, ice, and crank)?

Except the first two, response options for the other questions were not the same but were collapsed into comparable categories for analysis.

The mean number of days in the past 7 days that subjects engaged in moderate or vigorous physical activity was slightly higher among SHAPE app respondents compared with VCHS participants (Table 1). The median for all 3 physical activity items in the VCHS data was 1 day less than that in the SHAPE app data. Compared with the 2014 VCHS data (Table 2), fewer app respondents indicated smoking cigarettes in the past 30 days (11/143, 7.7% vs 33/206, 16%) and more app respondents selected the “never used” category (106/143, 74.1% vs 139/206, 67.5%).

The same percent of respondents in both surveys (11.9%, 17/142 and 11.7%, 24/205) selected the “never used” alcohol category (Table 2). VCHS subjects (Table 2) had a larger proportion of respondents, indicating alcohol use in the previous 30-day period compared with app respondents (76.1%, 156/205 and 69.0%, 98/142, respectively). The reference period for the binge drinking item was different in the 2 surveys—the previous 30 days for the app and the previous 2 weeks for VCH Survey. As noted in Table 1, app respondents had a higher mean (1.87) compared with VCHS (1.02). Given the longer reference period (30 days vs 2 weeks), it is reasonable to expect an increase in mean days reported for app respondents.

**Table 1:** Behavioral health characteristics of Castleton SHAPE app participants compared with results from the Vermont College Health Survey (VCHS) administered in 2014.

Physical activity, past 7 days	2016 Castleton SHAPE app respondents (n=414 survey 1, n=143 survey 2)			2014 Castleton VCHS participants (n=209)	
	Mean (SD)	Median	Mean (SD)		Median
Moderate-intensity exercise for at least 20 min (number of days)	3.11 (2.38)	3	2.56 (2.18)		2
Vigorous-intensity exercise for at least 20 min (number of days)	2.54 (2.41)	2	1.90 (1.98)		1
Performed exercises to strengthen muscles (number of days)	1.88 (2.07)	2	1.71 (2.01)		1
Number of times had 5 or more drinks of alcohol at a sitting (SHAPE last 30 days, VCHS last 2 wk)	1.87 (2.67)	0	1.02 (1.58)		0

**Table 2:** Behavioral health characteristics of Castleton SHAPE app participants compared with results from the Vermont College Health Survey administered in 2014.

Characteristics		2016 Castleton SHAPE app respondents, n (%)	2014 Castleton Vermont College Health Survey participants, n (%)
**Frequency of cigarette smoking, n (%)**			
	Never used	106 (74.1)	139 (67.5)
	Used, but not in last 30 days	26 (18.2)	34 (16.5)
	Used in last 30 days	11 (7.7)	33 (16.0)
**Frequency of alcohol use, n (%)**			
	Never used	17 (12.0)	24 (11.7)
	Used, but not in last 30 days	27 (19.0)	25 (12.2)
	Used in last 30 days	98 (69.0)	156 (76.1)
**Frequency of marijuana use, n (%)**			
	Never used	53 (37.1)	108 (52.7)
	Used, but not in last 30 days	47 (32.9)	42 (20.5)
	Used in last 30 days	43 (30.1)	55 (26.8)
**Frequency of cocaine use, n (%)**			
	Never used	129 (90.8)	191 (92.7)
	Used, but not in last 30 days	10 (7.0)	8 (3.9)
	Used in last 30 days	3 (2.1)	7 (3.4)
**Frequency of methamphetamine use, n (%)**			
	Never used	142 (100)	199 (97.5)
	Used, but not in last 30 days	0 (0)	4 (2.0)
	Used in last 30 days	0 (0)	1 (0.5)

**Figure 2:**
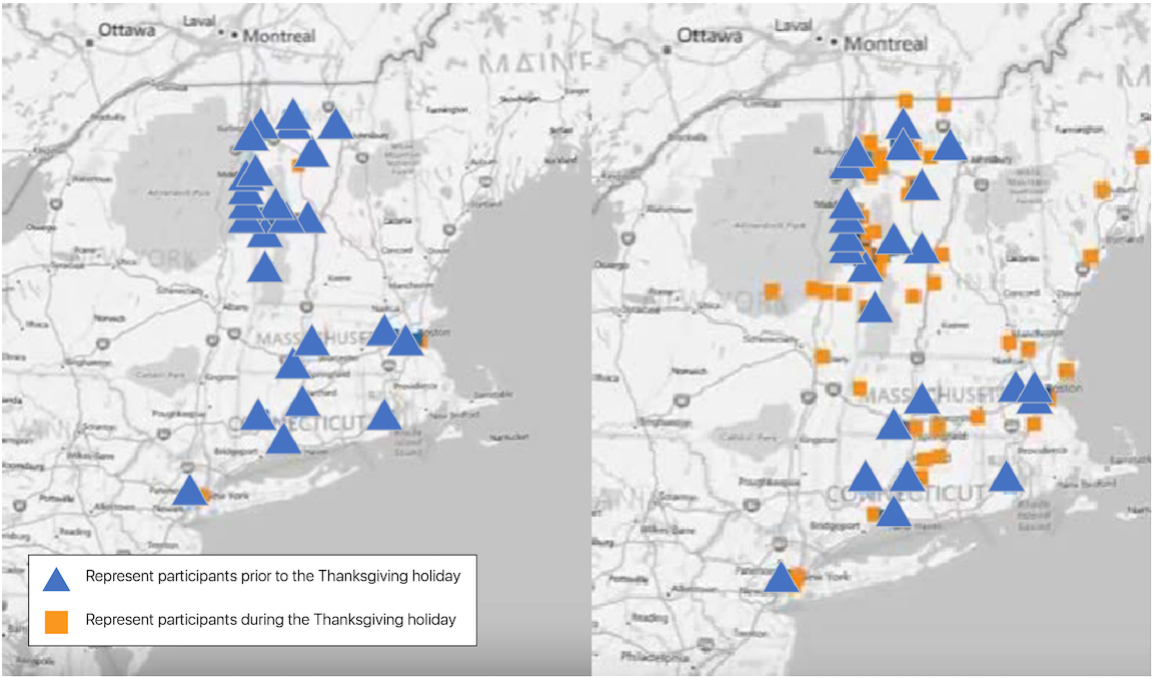
Geolocation data before and during Thanksgiving holiday.

A larger proportion of app respondents (Table 2) reported marijuana use than VCHS respondents (90/143, 62.9% and 97/205, 47.3%, respectively). The pattern in responses for cocaine use between the 2 datasets is similar with slightly lower frequency of “never” and “last 30 day” users among app respondents and a slightly higher rate in the “used but not in the last 30 days” category (10/142, 7.0%) compared with 3.9% (8/206) in the VCHS results. All respondents indicated that they had “never used” methamphetamine in the app compared with 97.5% (199/204) in VCHS.

### Acceptability: Paradata Findings

For Survey 1, 38.3% (176/459) of respondents completed the survey when the app was launched. Another 30.3% (139/459) completed it after the third push notification. For Surveys 2 and 3, the highest survey completion rate came with the arrival of the first reminder email. The overall time to project completion was an average of 37.6 hours for Survey 2 and an average of 11.6 hours for Survey 3. Nearly 90% of users (400/459, 87.1%) completed Survey 1 in <5 minutes with the median and modal response time of only 2 minutes. Surveys 2 and 3 were completed in 1 minute or less by the overwhelming majority of respondents.

Ambient data, such as geolocation, were easily captured for all users. There were 3 large data clusters centered near the 3 institutions that participated in the study. Two smaller clusters appeared in Manhattan and Connecticut during Survey 1, but these were likely nonglobal positioning system information. Survey 2 was fielded over the Thanksgiving holiday. By utilizing the geolocation data, we could see how much more dispersed the physical location of respondents was pre- and postholiday (Figure 2). Each triangle on the map indicates the geolocation of a survey respondent before the Thanksgiving holiday (diagram on the left) and each square represents the respondent during the Thanksgiving holiday (diagram on the right) with the before triangle also displayed. A few students appeared to have left “early” for the holiday because a few squares can be seen in the diagram on the left.

### Feasibility and Usability: Study Participant Feedback

In Survey 1, respondents were asked, “How did you hear about this project” and were given a list of options to select all that applied. The most frequently selected category (54%) was from someone else, followed by 28% indicating a flyer on campus, 15% via email, 14% a flyer in the bathroom, 11% somewhere else, and 1% via Facebook.

In addition, respondents were asked an open-ended question, “What is the primary reason you decided to download this app?” Overall, 37.9% (174/459) of respondents’ responses were categorized as “told about it by someone” with the “altruistic motivation” category closely following with 35.1% (161/459) of responses. The “personal reward” category had 10.9% (50/459) of responses, “general interest/curiosity” had 10.0% (46/459), “other” 5.0% (23/459), and “marketing materials” was at 2.2% (10/459).

The final survey was designed to gather evaluation data about the app. Results indicated that the app experience was positive. The majority of respondents (122/153, 79.7%) indicated that they would prefer to participate in surveys with an app on their phone compared with other modes of survey participation. Respondents were asked, “How much do you think you should be paid for downloading the app and answering the survey?” The results indicated nothing or a small amount under US $5 is preferred. Furthermore, 28.1% (43/153) of respondents were categorized as “promoters” based on their response to the question “How likely would you be to recommend this app to a friend?”

### Usability: Focus Group Post App Follow-Up

Participants in a focus group and interviews held after the close of all 3 surveys frequently stated that the app was easy to use and was intuitive. Those who completed the surveys and those who only partly completed them expressed that they expected more frequent surveys. When asked about the number of questions per survey, participants were satisfied with the length. Nearly all respondents wanted, at least, the option of seeing the results of the surveys after completing them and understand how they “compared” with their fellow students.

## Discussion

### Principal Findings

In this study, we were successful in rapidly recruiting participants with an initial group of 536 eligible participants. Notably, the number of participants for CU on the behavioral risk items was 414 for Survey 1, almost double the 209 CU participants who completed the VCHS in 2014. The survey app resulted in higher than usually observed response rates for longitudinal surveys [34].

Multiple technical successes were achieved throughout the app development process, indicating excellent feasibility for rapid development. Guidelines for successful mobile app development were followed [26], including the involvement the study population in the development process through focus groups and interviews, iterative UI and UX, field testing, and postfield testing follow-up through user focus groups. Medable removed multiple barriers (cost, time, technical knowledge, and HIPAA requirements) as a rapid mobile app development tool and a backend platform focusing on data security.

In addition, we were able to capture GPS data from participants’ phones; this highlights the exciting future potential to include additional sensor data to add richness to a dataset instead of just relying on self-report. Geocoded information combined with health information could open up the potential for additional analyses, such as the impact of the place or the neighborhood where people live on physical activity, diet, and drug use. The neighborhood environment has been shown to have an additional and distinct effect from individual characteristics [35-37].

Participants in the postsurvey focus groups viewed the app as being easy to use, engaging, and low burden. However, this information was gathered from those who completed all 3 surveys, and we do not have data from participants who completed just Survey 1 or Surveys 1 and 2. In the future work, we plan to assess the experience at the end of each survey with a few simple questions.

This project successfully demonstrated that one-fifth (472/2,342) of a college population (CU) would download, consent and be deemed eligible to participate in the SHAPE app and that researchers could make the survey experience low burden while maintaining the validity that is comparable with more expensive and burdensome efforts. Once study subjects downloaded the app, they were impressed with the speed and ease of entering their survey responses. Every indication suggests that the data collected were of good quality because both the correspondence between the app survey responses and the available benchmark comparisons were nearly identical and there was a low item nonresponse in all surveys. A limitation of this work is that we were unable to compare raw VCHS data to our mobile app survey data because of limited release of the VCHS data and because the samples were not entirely independent (although approximately 50% of the college population would likely have graduated); as a result, we did not conduct specific statistical testing of means and proportions or other summary measures.

Although the population selected for this study is not representative of the general US population, it is a well-defined and enumerated population. The study population closely mirrored the entire college population on which this study was focusing. However, because this app survey mechanism relies on voluntary download, the requirement of downloading an app could potentially introduce a selection bias in other populations and deserves further study in other scenarios. In addition, multiple recruitment methods were used that required in-person efforts, which may be difficult in larger communities. Gamification, which was one of the controlled variables, was underdeveloped and did not result in any difference in the survey response. The mobile app survey mechanism that this project developed has great potential for future research but requires further evaluation of potential barriers in broad population groups, particularly those that may be difficult to reach through current telephone and paper-based methodologies.

Recruiting a broad community-based population sample of subjects to download an app and consent to a data collection protocol will require additional methodological investigation, particularly because it relates to branding and social media marketing. Ideally, we would like to identify additional strategies to increase engagement, uptake, and retention.

Behavioral health assessment surveys collected by smartphone apps have great potential; very few respondents were lost during the consent and registration processes following download and most were likely the result of addressable technical and log-in challenges. The ability of smartphone users to “turn-off” notifications may have resulted in lower participation numbers for Surveys 2 and 3 and should be addressed.

A Cochrane review assessing the equivalence of data gathered through smartphone apps compared with other alternative delivery modes found that apps did not affect the data equivalence as long as the clinical application of the survey questions, the intended frequency of administration, and the setting remained unchanged [38]. Future extensions of this methodology include the ability to capture passively collected background data, such as accelerometer, and other ambient information; this information could proactively inform health providers, provide optimal resource allocation at the state and national agency level, and personalized information for a wide variety of health needs and health improvement objectives such as exercise, weight, and sleep. Moreover, the SHAPE app could be rapidly adapted to include additional surveys and other health metric outcomes of interest in either the college population or the general community. A value of native mobile apps is the ability to easily update them and encourage additional health survey data gathering for building projects and new data.

### Conclusions

This paper describes developing and pilot-testing a mobile app to administer behavioral health-related surveys on iOS and Android platforms targeting college students in Vermont.

The three key findings are as follows:

1. It was feasible to engage a large proportion of the target study population to download the SHAPE app, complete consent and eligibility determinations, and complete behavioral risk survey items. The first survey was completed by 459 participants and retention without incentives or other engagement tools was approximately 33% over time. Furthermore, the SHAPE mobile device survey system was very effective at including typically underrepresented groups.
2. Survey responses on behavioral risk items were valid because they were consistent with more expensive, larger survey efforts conducted using time-consuming methods. This paper compares CU app respondents with CU VCHS participants on 7 major behavioral risk items [27]. The data were valid and SHAPE was also deemed acceptable because it also captured ambient and real-time data that is not possible using conventional survey methods.
3. Mobile app survey systems can be used with low burden and quick response rates that includes ambient data such as geolocation. The average time for the overall project completion was 37.6 hours with an average of 11.6 hours from survey launch to completion response for Surveys 2 and 3, respectively. Once participants engaged in a survey, nearly 90% completed the survey in <5 minutes. Based on the data from questions in Survey 3 and postsurvey focus groups and interviews, participants found that the surveys were low burden, welcomed more engagement and questions, felt motivated by civic-mindedness, and were generally not concerned about being paid to participate. Moreover, students preferred to do a survey through an app on their phone compared with other modes of survey administration.

In summary, new techniques are emerging in survey methodology for public health and research. The private sector has moved ahead of the public sector on survey innovations. The internet and social media have become powerful methods of gathering information from consumers; voice recognition software allows businesses to offer rewards to consumers who call in to respond to surveys throughout the day. At the same time, participants are becoming resistant to cold calls in the evening as telemarketing has increased its pressure on the public. The goal of this pilot study was to determine if early adopters of smartphone technology would be likely to download an app to participate in a behavioral health survey. From this pilot study, we learned that the mobile app survey is a methodology that users of smartphone technology can employ with relatively low burden. The next step in the evolution of this technology and the methodology is to test this on a broad general population. This method alone will likely not be the sole means of collecting general population health data but rather a supplement. Currently, there is no one mode of data collection that can be used to conduct general population surveys. Even in-person only studies, which are prohibitively expensive for most researchers and government agencies, are limited in capturing data during daily life activities and suffer from erroneous self-report for certain sensitive information. As public health research evolves, leveraging current technologies to supplement data collection modes will be essential to capture rich and meaningful datasets and address nonresponse bias and coverage issues. The mobile app titled “SHAPE” developed in this project could be utilized in the future with the rapid addition of new surveys and new health content areas either in the college population or the general population in specific communities when warranted.

## Abbreviations

CU: Castleton University
HIPAA: Health Insurance Portability and Accountability Act
JSC: Johnson State College
LSC: Lyndon State College
RDD: random digit dialing
VCHS: Vermont College Health Survey
UI: user interface
UX: user experience
